# Non-Contact Water Level Response Measurement of a Tubular Level Gauge Using Image Signals

**DOI:** 10.3390/s20082217

**Published:** 2020-04-14

**Authors:** Sung-Wan Kim, Dong-Uk Park, Bub-Gyu Jeon, Sung-Jin Chang

**Affiliations:** Seismic Research and Test Center, Pusan National University, Yangsan 50612, Korea; kwenry@pusan.ac.kr (D.-U.P.); bkjeon79@pusan.ac.kr (B.-G.J.); sjchang@pusan.ac.kr (S.-J.C.)

**Keywords:** liquid storage tank, sloshing behavior, tubular level gauge, image enhancement, water level response

## Abstract

The occurrence of excessive fluid sloshing during an earthquake can damage structures used to store fluids and can induce secondary disasters, such as environmental destruction and human casualties, due to discharge of the stored fluids. Thus, to prevent such disasters, it is important to accurately predict the sloshing behavior of liquid storage tanks. Tubular level gauges, which visually show the fluid level of a liquid storage tank, are easy to install and economical compared to other water level gauges. They directly show the fluid level and can be applied for various fluids because they can be constructed with various materials according to the fluid characteristics and the intended use. Therefore, in this study, the shaking table test was conducted to verify the validity of the method for measuring the water level response of the tubular level gauge installed on a liquid storage tank using image signals. In addition, image enhancement methods were applied to distinguish between the float installed in the tubular level gauge and the gray level of the background.

## 1. Introduction

With the recent development of different industries, an increasing number of liquid storage facilities are being used in various industrial sites and public facilities, and the importance of facility management is increasing. Among such facilities, liquid storage tanks have been widely used as large-scale fuel and oil storage tanks in various industrial facilities and power plants, and as small-scale water tanks for domestic water and cooling or heating air-conditioning systems. Therefore, liquid storage tanks must maintain their functions in natural disasters. Failure to secure their safety may result in serious economic damage [1].

The damage to liquid storage tanks by dynamic loads such as earthquakes varies depending on the tank geometry, the materials used, and the types of stored fluids. Such damage is determined by the magnitude of the seismic load and the dynamic characteristics of the liquid storage tanks. Thus, to evaluate the safety of liquid storage tanks, it is important to identify their dynamic characteristics. Many studies have been conducted to identify the dynamic characteristics of liquid storage tanks, and it was found that their dynamic characteristics are affected by their geometry and materials, the types of fluids stored in them, their water level, and the excitation force applied on them [2,3,4,5]. When dynamic loads such as earthquakes are applied to a liquid storage tank, the hydrodynamic pressure acting on the structure and the dynamic behavior of the structure can be significantly amplified due to the interaction between the structure and the liquid inside it. This occurs due to the influence of the dynamic load caused by the sloshing of the fluid inside the liquid storage tank, and may seriously compromise the structural safety. Therefore, it is important to accurately identify the response of the liquid storage tank by considering the sloshing behavior of the fluid.

Research on the dynamic analysis of a liquid storage tank was conducted based on an analysis model, in which the linear theory was applied by assuming that the liquid is an incompressible ideal fluid and the tank is a rigid body. In the Niigata and Parkfield earthquakes, however, the tanks that were designed by assuming that a structure is a rigid body were significantly damaged by seismic loads. Therefore, the research on the behavior of liquid storage tanks has led to the development of analysis models that consider the relationship between the structure and the fluid, as well as the multidimensional non-linear behavior of the fluid. In particular, it was found that the influence of the secondary sloshing mode must also be considered, as damage was caused to large liquid storage tanks through sloshing due to the long-period seismic events in the Tokachi-oki earthquake [6]. Analysis methods that consider the ground, structure, and fluid–soil–structure interactions have been developed of late in response to the increasing demands for liquefied natural gas (LNG) storage facilities. The analysis models and methods developed in such studies can be verified using various methods, but they need to be verified through the shaking table test to more accurately reflect the dynamic behavior of fluids.

Studies were also conducted to analyze the effects of reduction devices on the sloshing behavior by considering only the horizontal vibration of liquid storage tanks, and to evaluate the effectiveness of the theoretical damping models [7,8,9]. In addition, there was a study in which the shaking table test was conducted using model water tanks, such as cylindrical, rectangular, conical, and pyramid-shaped tanks, and the results of the study were compared with those from finite-element analysis of the sloshing behavior of liquid storage tanks with various geometries [10]. A model water tank was fabricated to analyze the sloshing behavior of rectangular vessels in nuclear power plants under vertical seismic loads, and the shaking table test was conducted after infrared sensors were installed on top of the tank to measure the water level response of the fluid surface [11].

For the analysis of the sloshing behavior of fluids, measuring the water level response is basically required. Many studies have been conducted to analyze the sloshing behavior of the fluids inside liquid storage tanks caused by dynamic loads, such as seismic loads. In one study, the shaking table test was conducted to classify the resonant sloshing behavior of the liquid in a rectangular water tank and to verify the theoretical model, where the sloshing water level was measured using a wave gauge [12]. In another study, the shaking table test was conducted after installing a sloshing reduction device for the horizontal and vertical directions in an acrylic model tank, where the sloshing water level was measured using an ultrasonic sensor [13]. The same test was conducted in another study to experimentally verify the validity of the consistent particle method for the numerical simulation of sloshing, where the sloshing behavior was measured using a wave gauge and a water pressure gauge [14]. In general, sensors used to measure the water level response may malfunction due to factors such as water pressure and dynamic load, while measurement errors may occur due to the various external factors of the fluid surface [15]. Therefore, methods for simply and economically measuring the water level response of a liquid storage tank are required.

The use of non-contact methods for measuring the water level response using images is expected to increase [16], and many studies have been conducted to develop methods and algorithms to measure the water level response of liquid storage tanks. As for the related studies, a study proposed the method of using modified plane-based camera calibration to measure the surface waveform of a flume tank using a charge-coupled device (CCD) camera [17]. The method exhibited small errors compared to the wave gauges and ultrasonic water level gauges, and was proposed as an effective tool for measuring dynamic characteristics. In another study, the unidirectional shaking table test was conducted after fabricating a miniature model of a liquid storage tank and the sloshing behavior of the fluid was measured using a high-definition (HD) camera [18]. The sloshing behavior was measured in another study using an HD camera to experimentally evaluate the effect of the sloshing reduction devices used in steel storage tanks, which are commonly used in petrochemical complexes and oil storage facilities [19]. The 3D sloshing surface of the liquid in a water tank was measured in a small-scale shaking table test in another study by applying the image processing technique to the liquid [20]. A study was conducted to measure and compare the sloshing load and water level response of the liquid in a model water tank using an ultrasonic sensor and a digital camera [21]. A superconducting magnesium diboride (MgB2) water level gauge was installed in a study to measure the water level response of a liquid hydrogen storage tank in the shaking table test. The sloshing water level response was measured by installing an ultra-high-speed camera on the outside of the transparent tempered glass wall of a storage tank, and the results were compared with those measured using a water level gauge [22]. In addition, image filter processing was applied in a study to reduce the noise generated by the reflection of light on the fluid inside a circular liquid storage tank and to facilitate the recognition of control points in the shaking table test. There were small differences between the water level response to which image filter processing was applied and that measured using a water level gauge [23]. There were also studies that measured the velocity of fluids using particle image velocimetry (PIV) and particle tracking velocimetry (PTV). Liquid storage tanks must be fabricated using transparent materials to use PIV and PTV, which have been widely used to measure the water level responses of fluids. Inputting special particles in the tanks must be possible and the particles must be illuminated with a laser beam [24,25].

Meanwhile, tubular level gauges, which visually show the water level using Pascal’s principle or the principle of transmission of fluid pressure, are easy to install and economical compared to other water level gauges. As it directly shows the fluid level, it is easy to read. It can also be applied to various fluids because the tube can be constructed using various materials, such as glass and propylene, according to the fluid characteristics and the intended use.

In this study, a tubular level gauge was installed on the outside of a liquid storage tank, and a float was installed in the gauge. The water level response due to the movement of the float was measured using a digital camcorder and image enhancement methods were applied to distinguish between the float and the gray level of the background. Therefore, in this study, the shaking table test was conducted to verify the validity of the method used to measure the water level response of the tubular level gauge installed on a remote liquid storage tank using image signals.

## 2. Water Level Response Measurement Using Image Signals

### 2.1. Image Correlation Method

**Figure 1:**
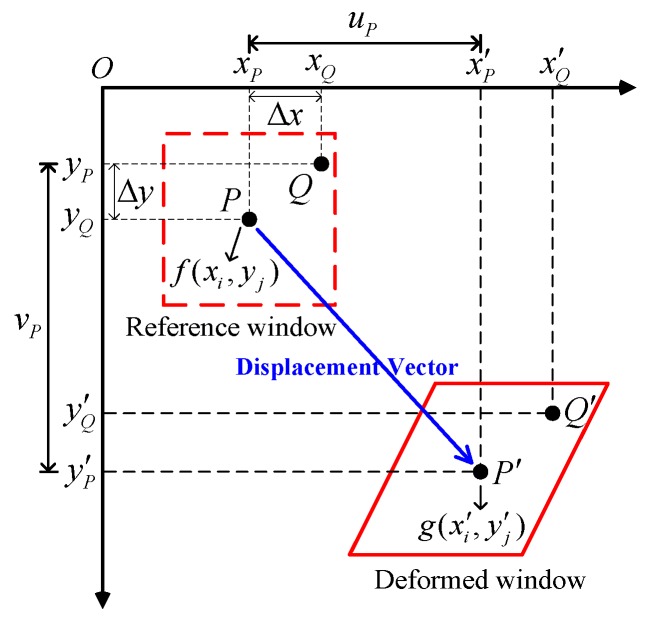
Deformation measurement using the image correlation method.

The image correlation method [26,27] is a non-contact optical measurement method that finds the displacements and strains of the control points that occurred due to the change in the target structure caused by an external force, by analyzing the correlation between the images before and after the deformation. The method uses the gray levels of the pixels in the images and reconstructs them into an array represented by numbers. It also measures the deformation by analyzing the correlations between the pixels in the images before and after the deformation. As deformation cannot be measured by analyzing correlations based on only unit pixel, a small square image referred to as a window is defined and used. The window represents a (2M+1)(2M+1) group or area. The window separated from the image before the deformation is referred to as the reference window. The control point, which is the center of the reference window, is the measurement point for the deformation of the structure caused by an external force. For the analysis of the correlation with the reference window, a window is separated from the image after the deformation, and it is referred to as the deformed window. A displacement vector can be obtained while the correlation between the control point of the reference window and that of the deformed window is being analyzed, and the deformation of the object by an external force can be measured using the displacement vector, as shown in Figure 1. In this study, the normalized cross-correlation (NCC) method in Equation (1) was used to anal yze the correlation between two images. The coordinates with the maximum value represent the displacement of the object caused by an external force. In Equations (1) and (2), $f(x_{i},y_{j})$ and $g({x^{\prime }}_{i},{y^{\prime }}_{j})$ represent the gray level values of the windows before and after deformation, respectively.

(1) $$C_{NCC}=\sum_{i=-M}^{M}\sum_{j=-M}^{M}\left[\frac{f(x_{i},y_{j})g({x^{\prime }}_{i},{y^{\prime }}_{j})}{\bar{f}\;\;\bar{g}}\right]$$

(2) $$\bar{f}=\sqrt{\sum_{i=-M}^{M}\sum_{j=-M}^{M}{\left[f(x_{i},y_{j})\right]}^{2}},\;\;\;\bar{g}=\sqrt{\sum_{i=-M}^{M}\sum_{j=-M}^{M}{\left[g({x^{\prime }}_{i},{y^{\prime }}_{j})\right]}^{2}}$$

For a general solid object, the influence of the resolution on the unit pixel is large when Equation (1) is applied. Therefore, to calculate the unit pixel, the shape function [28,29] is used to predict the deformation of the surrounding points based on the displacement measured at each point. The shape function assumes that an arbitrary point $Q(x_{Q},y_{Q})$ around point $P(x_{P},y_{p})$ before the very small deformation of a solid is converted into $Q^{\prime }(x_{Q^{\prime }},y_{Q^{\prime }})$ after deformation, and is still around $P^{\prime }(x_{P^{\prime }},y_{P^{\prime }})$, as shown in Figure 1. In general, for measurement methods that use the image correlation method, zero- to second-order shape functions are used. For the structure, the second-order shape function of Equation (3) was applied with consideration the bending and non-linear behavior [30]. In Equation (3), $\xi (x_{i},y_{i})$ denotes the shape function of the x coordinate, and $\eta (x_{i},y_{i})$ denotes the shape function of the y coordinate; $\Delta x=x_{P}-x_{Q}$ and $\Delta y=y_{P}-y_{Q}$, which represent the distance from point P to point Q, hold; u and v are the displacements at the window center position calculated through the image correlation method; M represents the size of the lattice point in the image before deformation, for which the displacement is to be measured; $u_{x}$, $u_{y}$, $v_{x}$, and $v_{y}$ represent the first-order displacement gradients; and $u_{xx}$, $u_{xy}$, $u_{yy}$, $v_{xx}$, $v_{xy}$, and $v_{yy}$ represent the second-order displacement gradients.

(3) $$\begin{array}{l} \xi_{2}(x_{i},y_{j})=u+u_{x}\Delta x+u_{y}\Delta y+\frac{1}{2}u_{xx}\Delta x^{2}+\frac{1}{2}u_{yy}\Delta y^{2}+u_{xy}\Delta x\Delta y\;\;\;(i,j=-M:M)\\ \eta_{2}(x_{i},y_{j})=v+v_{x}\Delta x+v_{y}\Delta y+\frac{1}{2}v_{xx}\Delta x^{2}+\frac{1}{2}v_{yy}\Delta y^{2}+v_{xy}\Delta x\Delta y \end{array}$$

### 2.2. Image Enhancement

The purpose of applying the image enhancement method [23,31,32] to an image is to convert the image for a special application. In this study, image enhancement methods in the spatial domain were applied to measure the response by distinguishing between the structure and the background, which have similar gray levels.

The averaging filter determines the average light intensity of the neighboring points defined by the filter mask, and reduces the transitions in light intensity by replacing the values of all the pixels in an image. As random noise is generally composed of distinct transitions in light intensity, an averaging filter is used to reduce the noise [33,34]. Therefore, the purpose of using an averaging filter is to reduce the random noise instead of decreasing the sharpness of the image and to connect the disconnected edges of the pixels. In this study, the median filter shown in Equation (4) was used to reduce the random noise generated by the non-linear behavior of the structure and to improve the discontinuous points. In Equation (4), R represents a median filter with the same row and column sizes, $x_{i}$ is the row size, and $y_{i}$ is the column size. Equation (4) is the average gray level of the pixels within the m×n neighbor points defined by the mask. All the coefficients of the filter are 1 instead of 1/mn. This is because having coefficients with a value of 1 is more efficient in terms of calculation. In the final filter processing step, the entire image is divided by m×n. A mask with an m×n size has a normalization constant equal to 1/mn, and is an averaging filter for which all the coefficients are identical.

(4) $$R=\frac{1}{m\times n}\sum_{i=1}^{mn}x_{i}y_{i}$$

The histogram of an image represents the distribution of bright or dark pixels. In the histograms of dark images with low light intensity and of bright images with high light intensity, the distribution of pixels is inclined to one side or concentrated at a specific position [35]. For an image with a concentrated pixel distribution, it is difficult to distinguish the structure from the background because the pixels have similar light intensities [36,37]. Therefore, in this study, the structure was distinguished from the background by performing histogram transformation to measure the response of the tubular level gauge. For the histogram transformation, histogram stretching [38,39] and gamma correction [40,41] of Equation (5) were applied. Equation (5) is a method of mapping [a, b] in the x section to [c,d] in the y section. Here, [a, b] represent the minimum and maximum light intensities of the original image, respectively, while [c,d] represent those of the transformed image. When γ is 1, identity transformation is performed. For γ<1, the output becomes brighter as the mapped value increases, and for γ>1, the output becomes darker because the mapped value decreases.

(5) $$y={\left(\frac{x-a}{b-a}\right)}^{\gamma }(d-c)+c$$

### 2.3. Algorithm Summary

**Figure 2:**
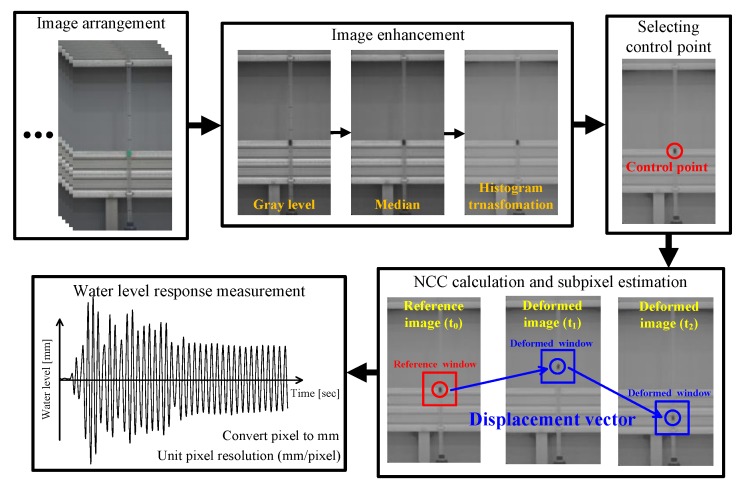
Algorithm used to estimate the water level response of the tubular level gauge using image signals.

Figure 2 shows the algorithm used to estimate the water level response of the tubular level gauge using image signals. The obtained video files are converted into image files and are arranged in chronological order. After the original images with RGB information are transformed into gray level images, image enhancement methods are applied to reduce the random noise, to improve the discontinuous points of the pixels, and to distinguish between the gray levels of the structure and the background. The user designates control points to the points in the reference window whose responses are to be known. There is no limit to the number of points to be measured. The NCC is calculated to provide information on the optimal matching from the reference windows, for which each control point is the center, to the deformed windows. In addition, for the unit pixels, the positions of the pixels were rearranged using the second-order shape function to correct the geometric distortions and measure the pixel-based responses. The resulting image analysis showed a pixel unit. To convert this into an actual distance, it is necessary to measure the actual size of the unit pixel on the image. Based on the image of a structure with a known size, the resolution of the unit pixel is obtained by dividing the size of the structure by the number of pixels to obtain the actual displacement. In this study, the algorithm described above was coded into an automated software program using MATLAB.

## 3. Estimation of the Water Level Response of the Tubular Level Gauge

### 3.1. Experiment Setup

**Figure 3:**
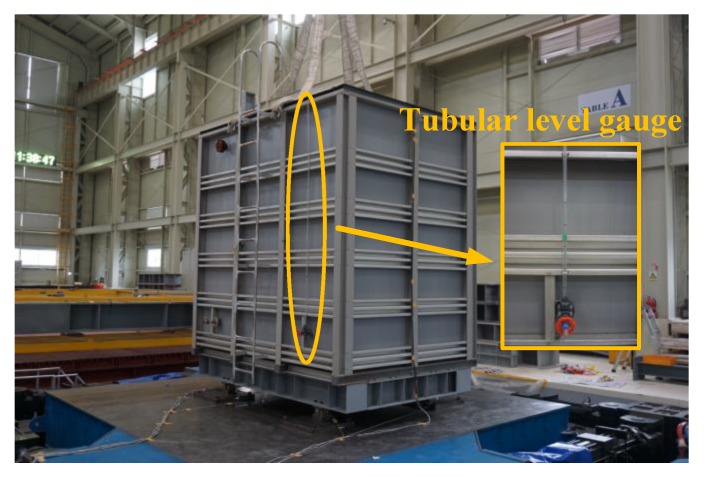
Specimen installed on the shaking table.

**Figure 4:**
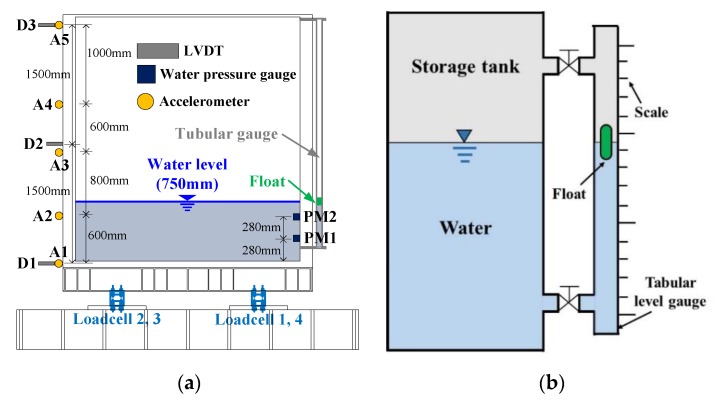
Installed sensors and principle of the tubular level gauge: (**a**) installed sensors; (**b**) principle of the tubular level gauge.

Although the liquid storage tanks used in buildings and general industries are made of various materials, the shaking table test was conducted on a polyethylene double-frame (PDF) tank, as such tanks are been widely used as digesters and liquid storage tanks in buildings. In general, the liquid storage tanks used in buildings or on building roofs do not have a standardized geometry due to the spatial characteristics of buildings. In this study, a 3 × 2 × 3 m (length × width × height) specimen was fabricated with consideration of the installation area, the behavioral characteristics of the fluid, and the limitations of the sensors to be installed on the shaking table. In addition, the water level of a liquid storage tank in a building may vary depending on the condition of the tank. In this study, however, more than 4 tons of water was stored inside the specimen. The shaking table test was conducted when the water level was 0.75 m. The ratio of the water level to the height of the specimen was 0.45. Figure 3 shows the specimen installed on the shaking table. Figure 4a shows the installation positions of the accelerometer, the linear variable differential transformer (LVDT), the water pressure gauges, and the load cell, as well as the tubular level gauge installed to measure the water level. Figure 4b shows the principle of the tubular level gauge. The pressure of the fluid is proportional to the density, gravitational acceleration, and water level, and the pressure intensity is transmitted in the same magnitude in all directions.

The tubular level gauge is generally installed on the outside of a fixed-type liquid storage tank. This shows the water level directly. For the tubular level gauge, an indicator is generally installed on one side of the tube, or a float is installed inside the tube for colorless liquids to increase the visibility of the water level. In this study, a float was installed inside the tube, as shown in Figure 4b, because the fluid that was used was a colorless liquid, and the water level response was measured using the movement of the float.

### 3.2. Dynamic Characteristics of the Liquid Storage Tank

**Figure 5:**
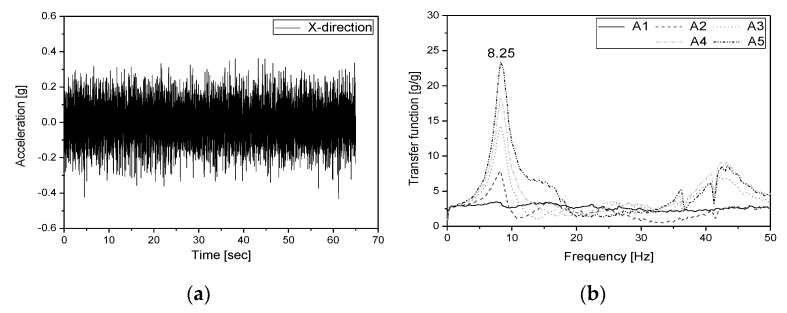
(**a**) Random wave and (**b**) transfer function.

**Figure 6:**
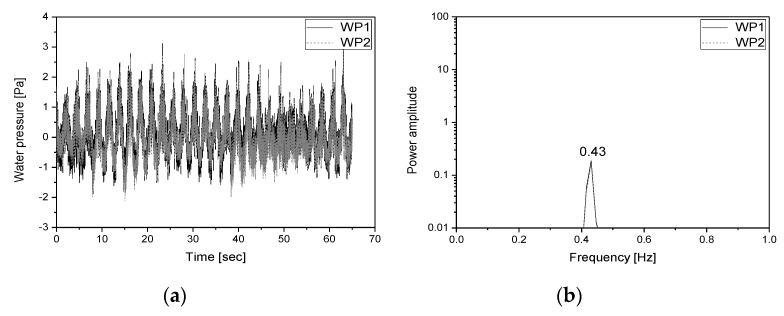
Water pressure response and power spectral density (PSD) function for such response (**a**) water pressure response; (**b**) PSD function for the water pressure response.

In this study, the dynamic characteristics of the specimen and the fluid inside the specimen were investigated using a random wave with wide frequency components (0.3–50 Hz). Figure 5a shows the input acceleration response for the shaking table. The first natural frequency of the specimen was estimated using the transfer function of the response measured from the installed accelerometers (A1–A5) to the input signal. Figure 5b shows the transfer function obtained from each accelerometer. The first natural frequency of the specimen was found to be 8.25 Hz. The natural frequency of the fluid inside the specimen was estimated using the frequency response obtained by applying the response signals of the water pressure gauges (WP1, WP2) to the power spectral density (PSD) function. Figure 6 shows the water pressure responses measured from the water pressure gauges and the PSD function for the responses. The first natural frequency of the fluid was found to be 0.43 Hz.

According to ACI 350.3 [42], the natural frequency of the convective component inside a rectangular liquid storage tank is determined by the height of the convective component and the length of the liquid storage tank in the direction of the applied acceleration, as shown in Equation (6).

(6) $$\omega_{c}=\frac{\lambda }{L}$$

(7) $$\lambda =\sqrt{3.16g\tanh\left[3.16\left(\frac{H_{L}}{L}\right)\right]}$$

The natural frequency of the convective component in a rectangular liquid storage tank can be obtained using Equation (6). In Equations (6) and (7), $\omega_{c}$ is the natural frequency of the convective component, L is the length of the water tank in the acceleration direction, $H_{L}$ is the height of the convective component, and g is the gravitational acceleration (9.806 m/s^2^). Factor λ is a function for the $H_{L}:L$ ratio. The acceleration direction that was applied in this study was the length direction. The internal length of the water tank was 2.9 m, excluding the wall thickness, while the water level was 0.75 m. The first natural frequency calculated using Equation (6) was 0.42 Hz, which was similar to the natural frequency of the first mode measured using the water pressure gauge (0.43 Hz).

### 3.3. Application of Image Enhancement Method

**Figure 7:**
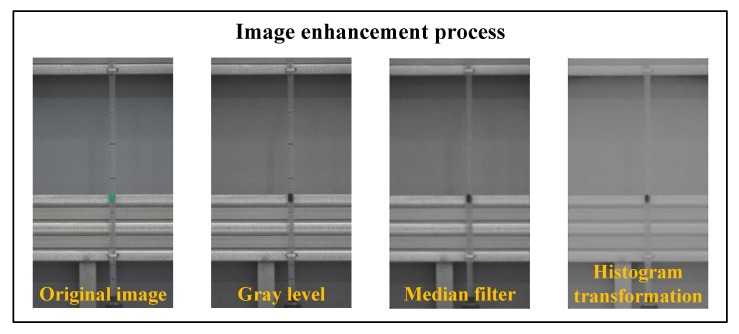
Processes of the image enhancement methods.

Figure 7 shows the image enhancement methods that were applied in this study. The video file acquired using a digital camcorder was converted into an image file (JPEG), which was the original image with RGB information. The original image was converted into an 8-bit gray level image for the image analysis. The median filter with a 21 × 21 mask was applied to the converted gray level image, and the image was subjected to histogram transformation. Double-type histogram transformation was performed. 0.3 and 0.6 were used for c and d in Equation (5). In this study, c and d, which are important parameters, were determined through trial-and-error learning so that the float installed inside the tubular level gauge could be clearly distinguished from the background of the structure. In addition, γ was set to 0.43, which was the average value of the double-type gray level in the gray level image.

**Figure 8:**
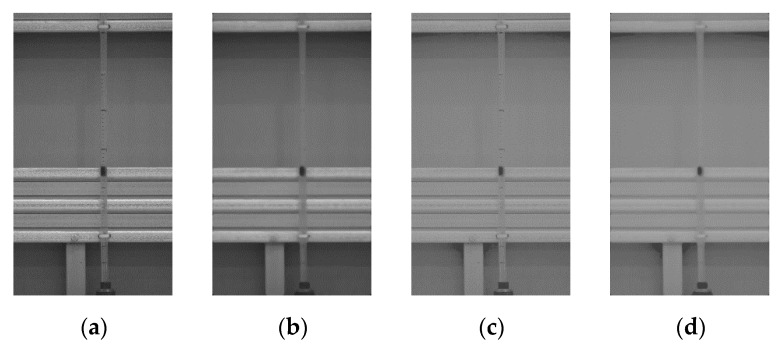
Images enhanced by the image enhancement methods (**a**) filter 1; **(b**) filter 2; (**c**) filter 3; (**d**) filter 4.

To verify the proposed tubular level gauge measurement algorithm, the water level response was measured for each algorithm presented in Table 1, the results of which are shown in Figure 8. Filter 1 comprised the gray level image converted from the original image with RGB information. Filter 2 comprised the image obtained by applying the median filter with a 21 × 21 mask to the gray level image. Filter 3 comprised the image acquired by applying histogram transformation to the double-type gray level image using c=0.3, d=0.6, and γ=0.43 in Equation (5). Filter 4 performed image analysis for the image obtained by applying the processes presented in Figure 7.

### 3.4. Water Level Response Measurement in the Shaking Table Test

In this study, the shaking table test was conducted to verify the validity of the algorithm used to measure the response of the tubular level gauge using image signals. For the image signals, video files acquired using a commercial digital camcorder (SONY FDR-AX40) were converted into images. For the video files, images with a 1920 × 1080 pixel size were stored at 60 frames per second and the frequency resolution Δf was set to 0.012 Hz. Before the shaking table test was performed, the resolution of the unit pixel was examined. In Figure 8a, the gap between the large scales drawn on the tubular level gauge is 100 mm and the corresponding number of pixels is 266. Thus, the resolution of the unit pixel is 0.375 mm/pixel. Therefore, the pixel-based displacement response measured by the water level response measurement algorithm presented in Figure 2 can be converted into an actual distance by multiplying it by the resolution of the unit pixel. Table 2 shows each load case for the shaking table test.

**Figure 9:**
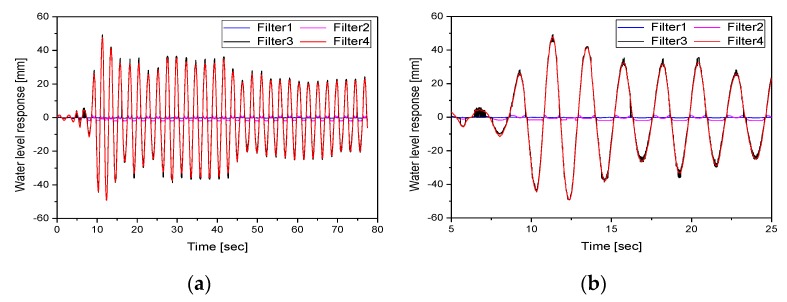
Comparison of the responses estimated by each image enhancement method in load case 2: (**a**) time history; (**b**) response between 5 and 25 s.

**Figure 10:**
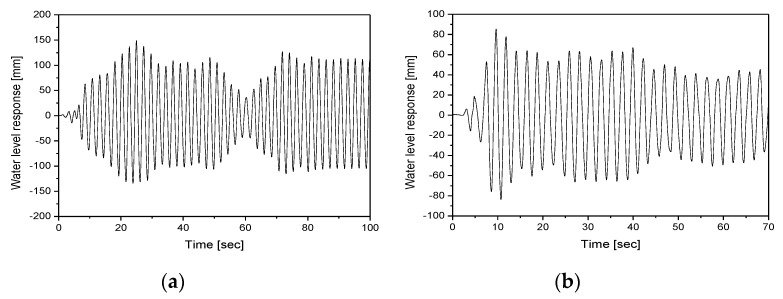
Responses in load cases 1 and 3 with filter 4: (**a**) load case 1; (**b**) load case 3.

In this study, the algorithm presented in Figure 2 was applied to extract the time history of the response to the control point of the float installed in the tubular level gauge. Figure 9 compares the responses estimated by applying each image enhancement method for load case 2. Figure 9a shows the time history and Figure 9b shows the responses between 5 and 25 s. Figure 9 shows that it was difficult for filters 1 and 2 to extract responses because the float installed in the tubular level gauge could not be distinguished from the background. In addition, the float installed inside the tubular level gauge exhibited non-linear behavior due to the pressure during the shaking table test. When image analysis was conducted using filter 3, discontinuity points, which occurred due to the influence of the scales drawn on the tubular level gauge and the random noise caused by non-linear behavior, caused an error in the recognition of control points. Filter 4 reduced the influence of the scales drawn on the tubular level gauge and the random noise caused by the non-linear behavior of the float using the median filter. In addition, the use of histogram transformation clearly distinguished the float installed in the tubular level gauge from the gray level of the structure, thereby facilitating the recognition of control points. Figure 10 shows the responses of the tubular level gauge in load cases 1 and 3 when filter 4 is applied.

(8) $$r_{xy}=\frac{\sum_{i=1}^{n}\left(x_{i}-\bar{x})(y_{i}-\bar{y}\right)}{\sqrt{\sum_{i=1}^{n}{\left(x_{i}-\bar{x}\right)}^{2}\sum_{i=1}^{n}{\left(y_{i}-\bar{y}\right)}^{2}}}$$

(9) $$Percent\;\;error=\frac{\sum_{i=1}^{n}{\left(x_{i}-y_{i}\right)}^{2}}{\sum_{i=1}^{n}{\left(x_{i}\right)}^{2}}$$

(10) $$RMS\;\;error=\sqrt{\frac{\sum_{i=1}^{n}{\left(x_{i}-y_{i}\right)}^{2}}{n}}$$

As no additional sensor was installed to verify the effectiveness of the proposed method, it is difficult to examine the accuracy and precision of the measured responses. Therefore, the validity of the proposed water level response measurement algorithm was verified by examining the similarity between the displacement response in the horizontal direction and the displacement response forced by the shaking table. In the proposed measurement method, which uses image signals, the displacement responses for the x and y axes are extracted. Therefore, it is possible to assume that the reliability of the y-axis response can be confirmed if the correlation coefficient of the x-axis response is high and the error is small. The cross-correlation coefficient of Equation (8) was used to examine the similarity between the displacement response forced by the shaking table and the response of the float extracted in the horizontal direction [23]. Equation (8) represents the correlation between two signals in the time domain. This is a function that is used to determine the similarity between the two signals. In addition, error analysis [31,43] was conducted on the displacement response forced by the shaking table and that of the float in the horizontal direction using the percent error of Equation (9) and the root mean square (RMS) error of Equation (10).

Here, $r_{xy}$ is the cross-correlation coefficient, x is the displacement response forced by the shaking table, $\bar{x}$ is the average of the displacement response forced by the shaking table, y is the response of the float in the horizontal direction, $\bar{y}$ is the average of the response of the float in the horizontal direction, and n is the number of measured data.

**Figure 11:**
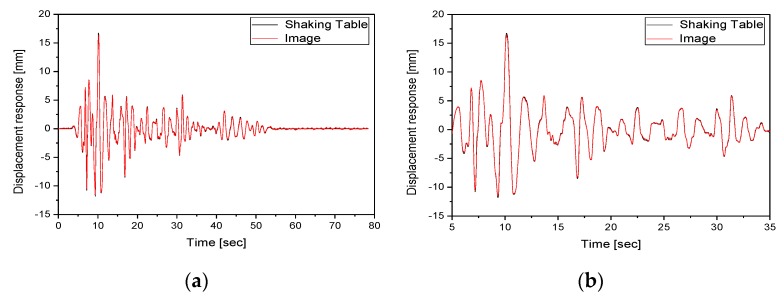
Comparison of the displacement responses in the horizontal direction in load case 2: (**a**) time history; (**b**) response between 5 and 35 s.

Figure 11 compares the displacement response forced by the shaking table with that of the float in the horizontal direction for load case 2. Figure 11a shows the time history, while Figure 11b shows the section between 5 and 35 s. Table 3 shows the cross-correlation coefficients and the results of the error analysis for the displacement response forced by the shaking table and that extracted using the image enhancement method in each load case. The cross-correlation coefficient has a value between −1 and +1. Here, +1 represents a complete agreement, −1 represents reversed phases with the same shape, and 0 represents no correlation. As can be seen in Table 3, the displacement response in the horizontal direction extracted using the image enhancement methods from filter 4 and that forced by the shaking table exhibited cross-correlation coefficients of 0.97 or higher, confirming the linearity of the two responses. In addition, there was a linear similarity between the two responses because they had high cross-correlation coefficients. The percent error was less than 1% and the RMS error was lower than 0.5 mm. This indicated that the displacement response forced by the shaking table and that of the float in the horizontal direction had high reliability. As the x-axis response extracted using image signals exhibited high reliability, the y-axis response was also found to be reliable.

**Figure 12:**
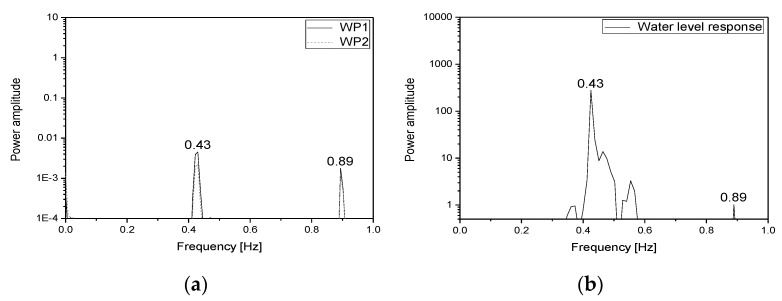
Comparison of the PSD functions of the responses measured in load case 2: (**a**) water pressure response; (**b**) image processing data.

Figure 12 shows the PSD functions obtained for the responses measured from the water pressure gauges and the response of the tubular level gauge estimated using the image processing data in load case 2. It was found that the natural frequency of each mode (first mode: 0.43 Hz; second mode: 0.89 Hz) had excellent reliability.

## 4. Conclusions

In this study, a non-contact measurement method based on image signals was proposed to measure the response of the tubular level gauge installed on a liquid storage tank during the shaking table test.

The error between the natural frequency of each mode obtained from the shaking table test by analyzing the image processing data and that of each mode obtained using water pressure gauges was less than 0.1%, thereby verifying the validity of the image processing data. In the shaking table test, the displacement response in the horizontal direction extracted using image processing and that forced by the shaking table exhibited cross-correlation coefficients of 0.97 or higher, confirming the linearity of the two responses. In addition, there were small errors between the two responses, as the percent error was less than 1% and the RMS error was lower than 0.5 mm. This confirmed the high reliability of the water level response extracted using image signals. It was also confirmed that the use of the proposed image enhancement method could reduce the error in recognizing the control points required for the measurement of the water level response and could facilitate measurement in cases where it is difficult to distinguish a structure from its background.

It was found that the proposed algorithm can be used when it is necessary to measure the water level response of a tubular level gauge in the shaking table test. For the long-term monitoring of the water level of a tubular level gauge in an externally located liquid storage tank, however, further research is required on the changes in lighting conditions and image noise.

## Figures and Tables

**Table 1:** Image enhancement methods used to measure the water level response.

Filter	Image Filter Processing
Filter 1	Gray level
Filter 2	Median
Filter 3	Histogram transformation
Filter 4	Median + histogram transformation

**Table 2:** Load cases of the shaking table test.

Load Case	Seismic Wave	Direction
1	Random	X
2	El Centro, 50%	X
3	El Centro, 100%	X

**Table 3:** Similarity and error analyses for each load case.

Load Case	Cross Correlation Function	Percent Error (%)	Root Mean Square (RMS) Error (mm)
1	0.988	0.427	0.162
2	0.983	0.359	0.141
3	0.979	0.545	0.258
